# Hospital Physicians’ Stethoscopes: Bacterial Contamination After a Simple Cleaning Protocol

**DOI:** 10.7759/cureus.37061

**Published:** 2023-04-03

**Authors:** Rachel L Dressler, Bonnie Cruser, Daniel D Dressler

**Affiliations:** 1 Department of Medicine, Emory University School of Medicine, Atlanta, USA; 2 Department of Medicine, Division of Hospital Medicine, Emory University School of Medicine, Atlanta, USA

**Keywords:** hospital medicine, microbial contamination, hospital cleaning, cleaning, stethoscope

## Abstract

Background: Stethoscope surfaces become contaminated with bacteria due to inconsistent cleaning practices,​ as​​ ​​​​cleaning frequency and practical clean​s​ing approaches are not well-established.

Methods: We investigated bacterial contamination of stethoscopes at baseline, after simple cleaning, and after examining one patient. We surveyed 30 hospital providers on stethoscope cleaning practices and then measured bacterial contamination of stethoscope diaphragm surfaces before cleaning, after cleaning with alcohol-based hand sanitizer, and after use in examining one patient.

Results: Only 20% of providers reported cleaning stethoscopes regularly. Before cleaning, 50% of stethoscopes were contaminated with bacteria, compared with 0% after cleaning (p<0.001) and 36.7% after examining one patient (p=0.002). Among providers who reported not cleaning stethoscopes regularly, 58% had bacterial-contaminated stethoscopes compared with 17% who did report cleaning regularly (p=0.068).

Conclusions: Hospital providers’ stethoscopes had a high probability of bacterial contamination at baseline and after examining one patient. We recommend decontamination with alcohol-based hand sanitizer immediately before each patient examination.

## Introduction

Stethoscopes are used in virtually every patient examination by hospital medicine physicians and hospital-based advanced practice providers (APPs). Several hospital-based studies have shown physicians’ stethoscopes are important vectors of infection, particularly via secondary transmission of infectious agents to healthcare providers’ hands or ​medical​​ ​equipment [1-6]. Youngster and colleagues found 85% of clinicians’ stethoscopes had colonization with *Staphylococcus* species-including 20% with methicillin-resistant *Staphylococcus* aureus (MRSA) [7]. Despite stethoscopes being known vectors for nosocomial transmitted infections, few guidelines direct or even address stethoscope hygiene standards for clinicians [8]. The guidelines that exist have poor adherence rates among providers [8,9], including students and trainees [10]. The Centers for Disease Control and Prevention (CDC) recommends cleaning stethoscopes after seeing each ​​​​patient [11], yet most physicians clean their stethoscopes infrequently (if at all), with a recent physician survey revealing more than half had never cleaned their stethoscopes [12]. 

While alcohol-based sanitizing solutions reduce bacterial contamination of stethoscopes [13], few physicians actually clean their stethoscopes in the setting of patient care; and anecdotal observations suggest that when stethoscopes are cleaned, that usually occurs ​after​ placing the device on a patient rather than before examination. Since stethoscope cleanliness (or bacterial contamination) after being used on just one patient has never been studied, we sought to determine bacterial contamination of stethoscopes a) directly from the hospital provider’s white coat pocket, b) after simple cleaning with alcohol-based hand sanitizer, and c) after being used to examine a single patient. We hypothesized that hospital providers’ stethoscopes before seeing patients and after seeing a single patient are more likely to be contaminated if not cleaned before each patient. 

## Materials and methods

We identified a convenience sample of unique inpatient providers (predominantly attending hospitalists, plus a small number of resident physicians and one APP) who cared for hospitalized patients (excluding those in the intensive care unit) at Emory University Hospital and Grady Memorial Hospital (Atlanta, Georgia). Each provider gave verbal consent to assess her/his stethoscope, and investigators recorded each provider’s personal estimated frequency of usual stethoscope cleaning practices​ and then​ collected​ ​sterile ​samples from each stethoscope. We defined regular cleaning of a stethoscope at least three times per day, as reported by the clinician. 

We applied a sterile swab application procedure to each stethoscope’s diaphragm surface to sample bacterial contamination at three ​discrete​ timepoints: 1) pre-cleaning (i.e., straight from doctor’s white coat); 2) post-cleaning (i.e., following 2-3 second investigator cleaning of the stethoscope diaphragm surface using ward-available alcohol-based hand sanitizer and a non-sterile paper towel, and after allowing the stethoscope to dry for 30 seconds); and 3) post-patient (i.e., after the clinician used her/his stethoscope to examine one [and only one] patient in routine fashion of patient care). Each of the three swabs of each stethoscope’s diaphragm surface was carefully and consistently applied to an individual pre​-​poured agar-plated Petri dish (Tryptic Soy Agar Plates, Evviva Sciences). A negative control Petri dish (sterile swab applied to agar plate without swabbing a stethoscope) and a positive control Petri dish (swab applied to agar plate after swabbing sole of a shoe worn in the hospital) were also incubated. 

Each swabbed and labeled Petri dish was incubated under a warming lamp at 80-90° Fahrenheit, allowing bacterial growth to occur over 96 to 120 hours (at which point, an image record was made of bacterial colony growth within the Petri dish). Those images were used to count the number of bacterial colonies of growth for each stethoscope’s pre-, post-, and one-patient swabbed Petri dishes. Each stethoscope served as its own “control” (i.e., the post-cleaning Petri dish served as control compared with the same stethoscope pre-cleaning or after one patient). We defined ​relevant ​stethoscope contamination as the growth of >3 bacterial colonies on a swabbed Petri dish. We did not evaluate specific types of bacteria that grew from the stethoscope swabs. Statistical analysis was performed using ​​simple Chi-Square and Fisher Exact Test analyses, as appropriate. 

## Results

Thirty clinicians consented to the assessment of their stethoscopes; one declined. By a survey report, only six of the 30 clinicians (20%) regularly cleaned their stethoscopes. 

**Figure 1:**
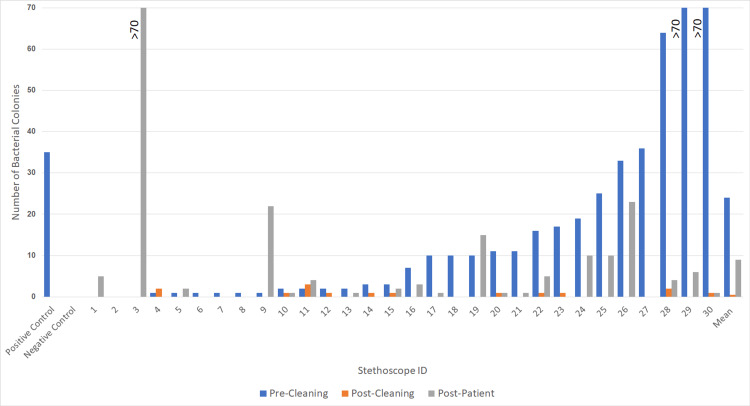
Bacterial colony count growth for each patient and mean: pre-cleaning, post-cleaning, and post-patient Post-patient = after examining a single (1) patient The mean number of bacterial colonies growing on stethoscopes pre-cleaning (mean 24.1 colonies), post-cleaning (mean 0.5 colonies), and post-patient (mean 9.0 colonies)

The negative control Petri dish plate had zero bacterial growth colonies (no contamination), and the positive control Petri dish plate had 35 bacterial growth colonies. Upon swabbing doctors’ stethoscopes straight from the lab coat pocket - i.e., pre-cleaning - stethoscopes averaged 24.1 bacterial growth colonies on their swabbed Petri dishes, and 50% of stethoscopes had contaminated bacterial growth pre-cleaning (Figure 1). 

Post-cleaning, stethoscopes averaged 0.5 bacterial colonies growth, and 0% of stethoscopes were contaminated (defined as >3 colonies) on their swabbed Petri dishes. Post-patient-after a clinician examined a single patient following the cleaning of the stethoscope-stethoscopes averaged 9.0 bacterial growth colonies, and 36.7% were contaminated after just one patient (Figure 1)​.​ 

**Figure 2:**
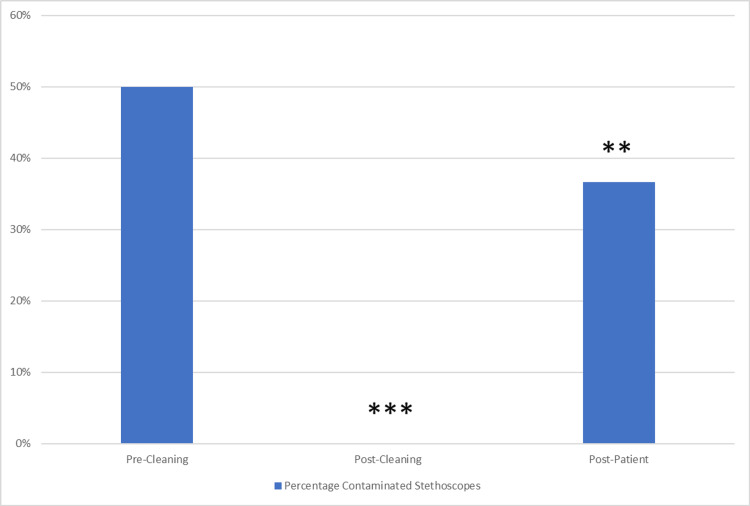
Percentage of contaminated stethoscopes pre-cleaning, post-cleaning and post-patient ***Comparison of analogous stethoscopes pre-cleaning to post-cleaning revealed a significantly greater percentage of bacterial-contaminated stethoscopes pre-cleaning:  50% pre-cleaning vs. 0% post-cleaning (p<0.001) **Comparison of analogous stethoscopes post-cleaning to post-patient (i.e., after 1 patient examined) revealed a significantly greater percentage of bacterial-contaminated stethoscopes post-one-patient:  37% post-patient vs. 0% post-cleaning (p=0.002)

Comparing analogous stethoscopes post-cleaning to pre-cleaning, there was a significantly greater proportion of bacterial-contaminated stethoscopes pre-cleaning (50% pre-cleaning vs. 0% post-cleaning, p<0.001, Figure 2).

**Figure 3:**
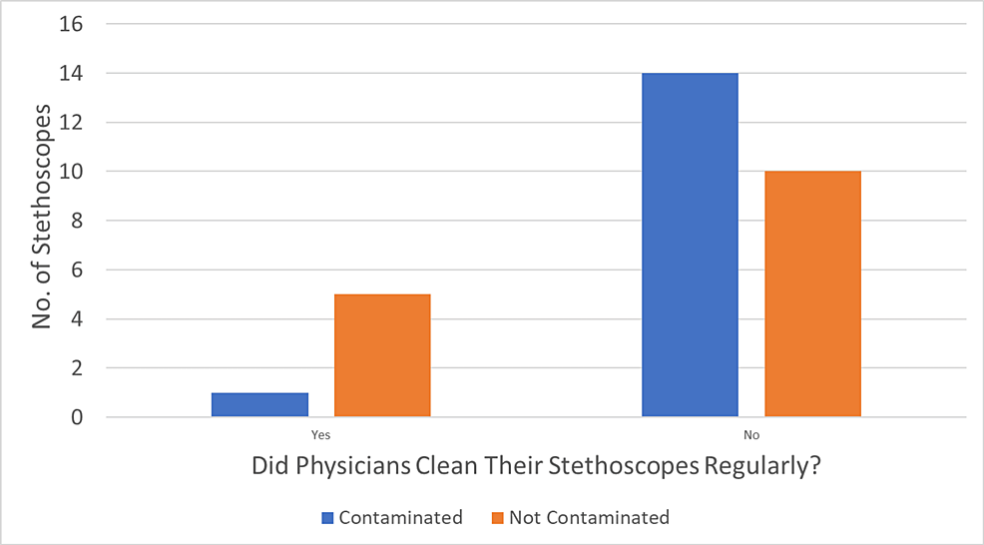
Stethoscope contamination (pre-cleaning) based on the clinician’s self-reported cleaning frequency Compared with clinicians who did not clean their stethoscopes regularly (answer “no” in the graph; defined as at least three cleaning episodes per day while using the stethoscope in the hospital), clinicians who did clean their stethoscopes regularly (answer “yes” in the graph) trended towards the lower likelihood of stethoscope contamination with bacteria straight from the clinician’s white coat (i.e., pre-cleaning; 58% vs. 17%, p=0.068).

Comparing analogous stethoscopes post-cleaning to those post-patient (i.e., after one patient), there was a significantly higher percentage of bacterial-contaminated stethoscopes after one patient (36.7% post-patient vs. 0% post-cleaning, p=0.002, Figure 2). Compared with clinicians who did not clean their stethoscopes regularly, clinicians who did clean their stethoscopes regularly trended towards a lower likelihood of stethoscope contamination with bacteria (pre-cleaning; 58% vs. 17%, p=0.068) (Figure 3).

## Discussion

In this small ​convenience ​survey of inpatient clinicians (predominantly practicing hospitalists), we confirmed our hypothesis that bacterial contamination of hospital providers’ stethoscopes before seeing patients (50% bacterial contamination rate) and after seeing a single patient (36.7% bacterial contamination rate) had a higher chance of being contaminated compared with stethoscopes cleaned before each patient (0% bacterial contamination rate). The vast majority (80%) of physicians in the study did not clean their stethoscopes regularly: 50% of stethoscopes, when swabbed straight from clinicians’ coat pockets, were contaminated with bacteria. A simple cleaning of the diaphragm surface using ​ward-accessible ​alcohol-based hand cleansing solution-readily available on all medical floors outside each patient room- could virtually eliminate bacterial contamination from the stethoscope surfaces.

Cleaning stethoscopes is a low-cost, low-time intervention that decreases bacterial contamination; further study can assess whether this, in turn, decreases the risk of nosocomial infection transmission and increases patient satisfaction. Although stethoscopes are noncritical items with less risk of primary infection transmission compared to invasive medical devices, it is plausible that they may contribute to secondary infection transmission via contamination of healthcare providers’ hands [2,3]. Previous research has linked MRSA colonization rates of stethoscopes with the colonization of healthcare providers’ hands, suggesting that the importance of cleaning stethoscopes may be comparable to that of cleaning hands [1]. The existing literature is clear that physician hand cleanliness is important to patients. One survey found that most patients would not want a physician to care for themselves or for their family members if the physician’s hands were not cleansed before touching anything in the room [14]. As performing other hygienic actions (e.g., handwashing) in the patient's view demonstrates cleanliness and increases their satisfaction and trust, regular stethoscope cleaning immediately before use is likely to do so as well.

In our study (and anecdotally with other providers), clinicians who clean their stethoscopes tend to do so after examining a patient; however, our findings suggest that cleaning should be ​performed​ ​immediately ​before examining each patient. One in six of the clinicians who regularly cleaned their stethoscopes still had bacterial contamination right out of the coat pocket, and 36.7% of the stethoscopes were contaminated with bacteria after seeing just one patient. For maximum effectiveness, stethoscopes must be cleaned ​before​ examining each new patient. 

This study has several limitations. First, when doctors went to see one patient, their stethoscope use was not directly observed; rather, they were simply instructed to do their usual ​stethoscope ​practice. Doctors thus could have cleaned their stethoscopes when they typically might not, introducing a possible Hawthorne effect [15]. Regardless, the presence of ​such​ bias would ​likely ​strengthen our findings​,​ as real-world rates of stethoscope cleaning are likely even lower than that observed ​in ​our study participants; additional study is needed to determine the clinical significance of stethoscope contamination. Second, patient medical conditions were unknown, and some patients could have ​clinical conditions with a ​greater likelihood to transmit bacteria to stethoscopes than others. However, participating clinicians--when using their stethoscopes on a single patient (for the after one patient measurement)--were instructed to examine only patients in non-isolation rooms. Third, our study was conducted with clinicians examining only hospital general ward patients, not patients in the ICU, and our results, therefore, may not extrapolate to clinicians caring for ICU patients. Nevertheless, we believe that the principle of stethoscope cleaning before each-and-every patient has no obvious downsides, can reasonably be applied across all inpatient clinician encounters, and is feasible for adoption by providers through education and job aids such as visible reminders [16,17].

## Conclusions

In conclusion, this study demonstrates that hospital providers’ stethoscopes had a high probability of bacterial contamination at baseline and after examining one patient; bacterial contamination can occur in as many as one-third of clinicians’ stethoscopes after they have seen only a single hospitalized patient. We also confirmed prior studies demonstrating cultural dissonance related to stethoscope cleaning practices by physicians, and our study suggests that clinicians who clean their stethoscopes regularly may be less likely to have contaminated stethoscopes even if they forget or miss a cleaning opportunity. We recommend decontamination with alcohol-based hand sanitizer immediately before each patient examination. Regular, simple stethoscope cleaning efforts before seeing patients could effectively reduce bacterial contamination of this commonly used equipment. Future studies should focus on types of organisms that grow on stethoscopes and clinical patient outcomes based on provider stethoscope cleaning practices. 
